# Valorization of Olive Pomace Using Ultrasound-Assisted Extraction for Application in Active Packaging Films

**DOI:** 10.3390/ijms25126541

**Published:** 2024-06-13

**Authors:** Renia Fotiadou, Ioanna Fragkaki, Kyriakos Pettas, Haralambos Stamatis

**Affiliations:** 1 Laboratory of Biotechnology, Department of Biological Applications and Technologies, University of Ioannina, 45110 Ioannina, Greece; p.fotiadou@uoi.gr (R.F.); ioannafr2@gmail.com (I.F.); 2 STYMON Natural Products ΙΚΕ, Industrial Area of Patras, Street B2, Building Square 4, 25018 Patras, Greece; domenico.pettas@stymon.com

**Keywords:** olive by-product, polyphenols, hydroxytyrosol, tyrosol, HPLC analysis, antioxidants, antibacterial activity, chitosan, PVA, UV block

## Abstract

Bioactive compounds that can be recovered by the solid wastes of the olive oil sector, such as polyphenols, are known for their significant antioxidant and antimicrobial activities with potential application in nutraceutical, cosmetic, and food industries. Given that industrial demands are growing, and the polyphenol market value is ever increasing, a systematic study on the recovery of natural antioxidant compounds from olive pomace using ultrasound-assisted extraction (UAE) was conducted. Single-factor parameters, i.e., the extraction solvent, time, and solid-to-liquid ratio, were investigated evaluating the total phenolic content (TPC) recovery and the antioxidant activity of the final extract. The acetone–water system (50% *v*/*v*, 20 min, 1:20 g mL^−1^) exhibited the highest total phenolic content recovery (168.8 ± 5.5 mg GAE per g of dry extract). The olive pomace extract (OPE) was further assessed for its antioxidant and antibacterial activities. In DPPH, ABTS, and CUPRAC, OPE exhibited an antioxidant capacity of 413.6 ± 1.9, 162.72 ± 3.36 and 384.9 ± 7.86 mg TE per g of dry extract, respectively. The antibacterial study showed that OPE attained a minimum inhibitory activity (MIC) of 2.5 mg mL^−1^ against *E. coli* and 10 mg mL^−1^ against *B. subtilis*. Hydroxytyrosol and tyrosol were identified as the major phenolic compounds of OPE. Furthermore, active chitosan–polyvinyl alcohol (CHT/PVA) films were prepared using different OPE loadings (0.01–0.1%, *w/v*). OPE-enriched films showed a dose-dependent antiradical scavenging activity reaching 85.7 ± 4.6% (ABTS) and inhibition growth up to 81% against *B. subtilis* compared to the control film. Increased UV light barrier ability was also observed for the films containing OPE. These results indicate that OPE is a valuable source of phenolic compounds with promising biological activities that can be exploited for developing multifunctional food packaging materials.

## 1. Introduction

Olive pomace (OP) is the main solid residue (approximately 80–85%) derived from the two-phase extraction of olive oil and constitutes 45% of the total wastes derived from the three-phase extraction of olive oil. This by-product consists of olive bodies, pulp, leaves, kernel, and pit fragments and its composition depends on the variety and cultivation of the olive trees, the extraction process, etc. The total phenolic content of OP is significantly high, exhibiting inevitable biotoxicity and phytotoxicity when it is disposed into the environment. Up to now, OP has been used as biofuel or animal feedstock in line with the current regulations, but the increasing demands for olive oil and related products make the amount of biowastes that are accumulated difficult to handle [1,2]. Meanwhile, the market of olive polyphenols is ever growing. This trend guides the exploitation of OP to meet the current demands as a low-cost source of valuable bioactive compounds with minimum environmental impact.

Polyphenols are aromatic antioxidant compounds that exhibit several biological activities (antioxidant, antimicrobial, cardioprotective, etc.). One of the main goals of the food, pharmaceutical, and nutraceutical industries is the replacement of synthetic antioxidants, such as butylated hydroxyanisole, butylated hydroxytoluene, and ethylenediaminetetraacetic acid, with natural phenolic compounds driven by the concerns of consumers [3]. Under that scope, the extraction of natural phenolic compounds from agro-industrial by-products is an ongoing strategy. Ultrasound-assisted extraction (UAE) is a green, non-conventional method applied to solid wastes. The main advantages of this process are the shorter extraction time, the lower solvent and energy requirements, and the ease of scale-up. Ultrasound waves induce disruption in the solid waste by creating physical forces during acoustic cavitation, which improve mass transport and facilitate the release of extractable components into the solvent. Moreover, the instant increase in temperature improves the solubility and diffusion of the phenolic compounds into the solvent. Meanwhile, GRAS solvents, such as water, ethanol, and acetone, are primarily used to prepare “food-grade” polyphenol extracts [1,4,5,6].

Among the bioactive compounds present in OP are oleuropein, hydroxytyrosol, tyrosol, luteolin, vanillic acid, etc. The concentration of each polyphenol depends on the variety and the maturation of olives, the extraction process, and the storage conditions. During the maturation of olives or under extraction processing, oleuropein hydrolyses to hydroxytyrosol [4]. Hydroxytyrosol is considered one of the most powerful natural antioxidants. Due to its catechol moiety, hydroxytyrosol exhibits enhanced free radical scavenging properties. The antioxidant, anti-inflammatory, cardioprotective, antibacterial, and antifungal activities have been extensively highlighted. As a result, the European Food Safety Authority has given recognition to these attributes, and so hydroxytyrosol is Generally Recognized as Safe. However, the production of functional foods or bioactive films is still a challenge due to the economic burden. The price of pure hydroxytyrosol is still high [7,8,9]. To address these issues, the optimization of polyphenols’ extraction from OP is of great interest. 

Polyphenols extend the shelf-life of foods by functioning mainly as antiradical and antimicrobial agents. Nowadays, active packaging has emerged as a new approach to preserve the quality and organoleptic properties of foods from degradation. Plant extracts are incorporated into polymer films to enhance their bioactivity [10,11]. Chitosan (CHT) is a natural polysaccharide that is widely used due to its green character and desired biological activities (antimicrobial) [12]. Polyvinyl alcohol (PVA) is a synthetic polymer with low production cost, flexibility, non-toxicity, biodegradability, and FDA approval for food packaging systems [13,14,15]. The polymer blending of natural and/or synthetic polymers is currently an effective way to prepare active films with combined properties and minimum cost [16]. Composite films of CHT, PVA or the blending of them with natural extracts as additives have been reported to display improved and desirable characteristics [16,17]. Many literature reports pursue the casting method for film preparation, owing to the lack of necessity for organic solvents, special equipment, and harsh conditions (e.g., heating temperature). Meanwhile, this approach supports the increasing endeavors to replace non-biodegradable films produced by conventional petroleum-based plastics.

In this study, we aimed for the recovery of polyphenols, with an emphasis on hydroxytyrosol and tyrosol, from OP using UAE. The objective of this work was to investigate the effect of four parameters (extraction solvent, water-to-organic solvent ratio, liquid-to-solid ratio, and extraction time) as a function of the TPC recovery and the antioxidant activity of OPE using a single-factor experiment approach. The final extract was fully characterized regarding the TPC, TFC, antioxidant (DPPH, ABTS, and CUPRAC), and antimicrobial activities, and the profile of polyphenols through HPLC analysis. Furthermore, active CHT/PVA films were prepared with the incorporation of different concentrations of OPE. The optical, structural and biological properties of the different films were evaluated. The antioxidant activity was estimated through the DPPH and ABTS assays, and the antimicrobial activity was screened against *E. coli* and *B. subtilis*. To the best of our knowledge, this is the first report where OPE is used as active ingredient of CHT/PVA coating films, exhibiting enhanced bioactivities and applying the theme of circular economy.

## 2. Results

### 2.1. UAE of Polyphenols from Olive Pomace

**Figure 1:**
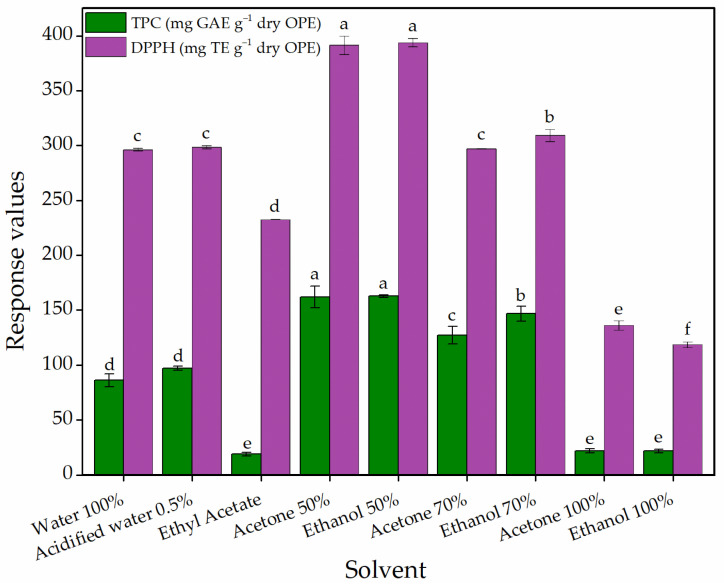
Effect of solvent on the total extraction of polyphenols from OP and the antioxidant activity. Different lowercase letters indicate differences in significance.

The choice of solvent is crucial in the extraction process regarding not only efficiency but also sustainability and economy. Ethanol, acetone, and ethyl acetate are among the widely used organic solvents for the recovery of bioactive compounds from plant-based by-products due to their low toxicity according to the European Food Safety Authority. A greener and more productive approach is the combination of these organic solvents with water, aiming to decrease the cost and the environmental impact of the emissions of the volatile solvents and also to improve the recovery yields. In this framework, the effect of different solvents in combination with ultrasound waves on the extractability of polyphenols from OP was studied as a function of the TPC recovery and the antioxidant activity of the dry OPE. As shown in Figure 1, the highest recovery was achieved when 50% acetone or 50% ethanol (*v*/*v*) were applied as extraction solvents. The TPC ranged from 161.8 ± 7.9 to 162.9 ± 1.3 mg GAE per g of OPE (*p* > 0.05) and the antioxidant activity from 391.4 ± 3.8 to 393.7 ± 3.3 mg Trolox per g of OPE (*p* > 0.05). The recovery yield of polyphenols and the DPPH radical scavenging activity of the extracts derived from these systems significantly differed from the other solvent systems (*p* < 0.05). Water–ethanol and water–acetone mixtures increased the recovery of polyphenols. Similar observations were reported by Gomez-Cruz et al. [18]. The polarity of the solvent system greatly affects the extraction process by increasing the solubility of the bioactive compounds in the medium [3]. In this respect, Soufi et al. [19] comprehensively elucidated the impact of the extraction solvent in combination with ultrasound waves on the effective release of phenolic compounds from OP. The role of acetone on polyphenols’ recovery from different by-products has been also highlighted by numerous works [20,21]. In the current study, the acetone–water mixture was chosen for further optimization experiments due to less related studies on the extraction of polyphenols from OP in combination with UAE.

#### Evaluation of Single-Factor Experiments

**Figure 2:**
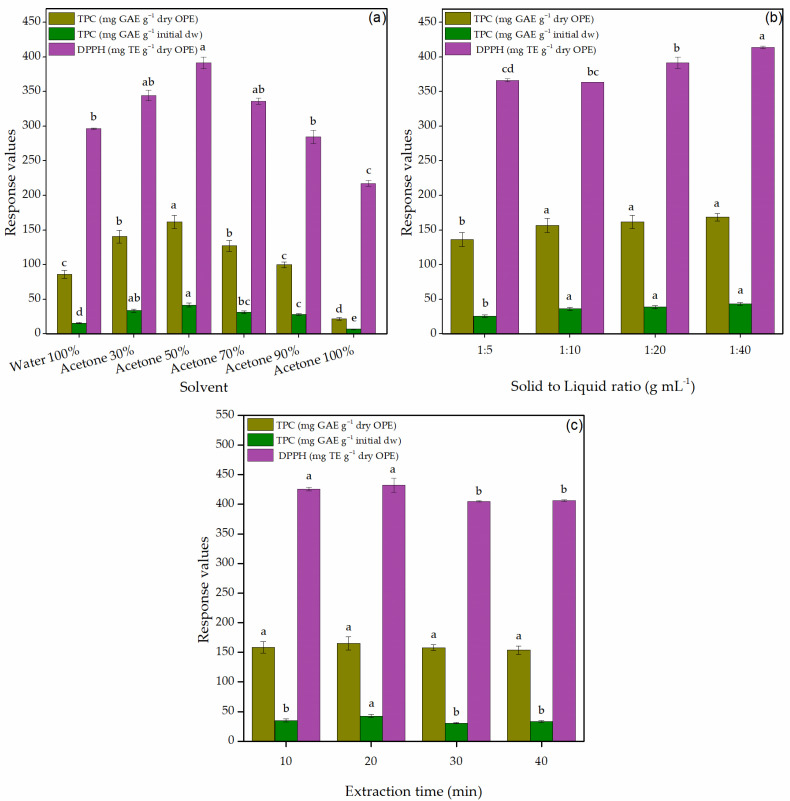
Effect of three single factors on the extraction of polyphenols from OP and the antioxidant activity: (**a**) acetone content (%), (**b**) solid-to-liquid ratio (g mL^−1^), and (**c**) extraction time (min). Different lowercase letters indicate differences in significance.

The acetone–water ratio was further examined as a parameter influencing the recovery of polyphenols. As shown in Figure 2a, the acetone–water ratio greatly affects the TPC recovery and the antioxidant activity of the final extract. This observation can be explained by the principle of similarity and intermiscibility. Specifically, when the solvent and the solute exhibit comparable levels of polarity, a higher recovery of phenolic compounds is attained. Notably, the utilization of a mixture of organic solvent and water as the extraction medium surpasses the efficacy of using a pure solvent in the extraction of phenolic compounds [1,19]. It is evident that 50% acetone (*v*/*v*) was the optimum, achieving a TPC recovery of 161.8 ± 7.9 mg GAE per g of OPE or 41.7 ± 2.9 mg GAE per g of initial dry weight (dw). Moreover, the antioxidant activity was 391.4 ± 3.8 mg Trolox per g of OPE. The results were statistically significant (*p* < 0.05). Soufi et al. [19] also found that the optimum value for polyphenols’ recovery from the Azeradj cultivar was 57.34% acetone (*v*/*v*).

The solid-to-liquid ratio was also investigated. As shown in Figure 2b, the TPC recovery and the antioxidant activity increased as the volume of the extraction solvent also increased. Specifically, the TPC (136.4 ± 10–168.8 ± 5.5 mg GAE per g of OPE or 25.8 ± 1.9–43.6 ± 2.0 mg GAE per g of initial dw) and the antioxidant activity (365.9 ± 2.3–413.6 ± 1.9 mg Trolox per g of OPE) were higher when the solvent ratio increased from 1:5 to 1:40. This can be attributed to an increase in the contact area between the solid material and the solvent, resulting in enhanced solubility and greater dissolution of the polyphenols into the solvent during the process of UAE. Similar findings have been documented for the extraction of polyphenols from other plant by-products [19,22,23]. Gertenbach [24] stated that mass transfer relies on the gradient between the solid material and the solvent. When the solvent-to-solid ratio is higher, there is a notable increase in the concentration balance between the polyphenols confined within the particles and the surface of the solid material. This favorable condition efficiently promotes mass transfer and expedites the extraction kinetics [25,26,27]. However, the TPC recovery from 1:10 to 1:40 ratios did not show any statistical significance in contrast to the results of the DPPH antiradical assay. Hence, the ratio 1:20 was used for further experiments to maintain low solvent requirements, which is also a major concern for downstream processing.

The extraction time has also a significant effect on the extractability and the quality of polyphenols. From 10 to 20 min, UAE showed an increasing TPC recovery and antioxidant efficacy, whereas a longer extraction process had negative results (Figure 2c). It is worth noting that as the extraction time increases, the degradation of polyphenols is also observed [28,29]. For those mentioned, UAE requires shorter process time compared with conventional techniques [30,31], ranging from 2 to 75 min [5,19] to reach the optimum recovery yields.

### 2.2. Total Characterization of the Optimized Extract

**Figure 3:**
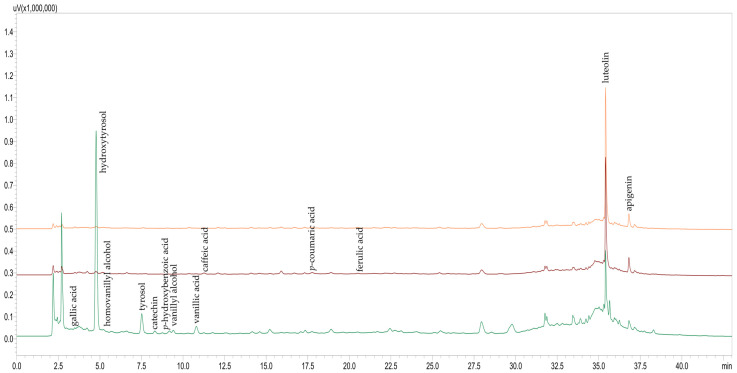
Representative chromatogram of acetone OPE at selected wavelengths (green: 280 nm, red: 320 nm, orange 346 nm).

Table 1 presents the profile of the phenolic compounds recovered from OP. In detail, fifteen known chemical components were identified and quantified using HPLC-UV analysis (Figure 3). Khairy et al. [4] also revealed a similar phytochemical profile. Hydroxytyrosol, tyrosol, catechin, luteolin, vanillyl alcohol, and vanillic acid were abundantly present in acetone OPE. Previous studies have also verified that hydroxytyrosol and tyrosol are the principle phenolic compounds recovered by UAE from OP [6,32]. In the present work, a high content of hydroxytyrosol was observed up to 48.47 ± 0.13 mg per g of extract under the optimized experimental conditions compared to 36 ± 2 mg per g of extract reported by Madureira et al. [6]. However, the aforementioned study revealed up to a 2-fold higher tyrosol concentration recovery. Cepo et al. [33] and Aliakbarian et al. [34] reported relatively lower hydroxytyrosol and tyrosol contents (expressed as mg per g of dry matter) but a higher oleuropein quantity. These differences can be attributed to the origin of the by-products, the pre-processing, and the extraction methodology. Acetone OPE was also compared with ethanolic OPE recovered under the same experimental conditions. As revealed by the HPLC analysis, acetone OPE was richer in specific phenolic compounds of interest, i.e., hydroxytyrosol, tyrosol, luteolin and catechin, but had a lower content of caffeic, p-hydroxybenzoic, and vanillic acid.

Table 2 shows the total phenolic and flavonoid content of the optimized OPE (UAE, 50% acetone, 20 min, 1:20). TPC is relatively higher per dw of the initial biomass compared to the recent literature reports employing similar (UAE, 57.34% acetone) or different extraction media (UAE, deep eutectic solvent with caffeic acid) [3,19]. Goldsmith et al. [5] and Cepo et al. [33] reported TPC values of 19.71 ± 1.41 mg per g of OPE using 100% water and 14.7 mg GAE per g of OPE using 60% ethanol, respectively. These findings are about eight times lower compared to this study. In our previous work, TPC recovery was 112 mg per g of olive leaf crude aqueous extract using the conventional maceration technique (1:10, 90 °C, 20 min) [35]. Regarding the TFC, Soufi et al. [19] reported a total recovery value of 10.08 g CE per kg of dry OP, which is five times higher compared to the results of this study using similar extraction conditions. These findings prove that UAE is among the most effective methods to recover polyphenols in mild conditions, requiring a short extraction time and producing high yields, and that the total biochemical characteristics of the final extract depend on the extraction parameters, specifically the solvent, and the origin/maturation/composition of the initial material. Acetone OPE was also compared with ethanolic OPE recovered under the same experimental conditions. As presented in Table 2, the TFC (mg QE g^−1^ dry extract) and the extraction yield (%) of acetone OPE were significantly different and, more specifically, higher than the corresponding values of the ethanolic OPE (*p* < 0.05).

The antioxidant activity of the optimized extract was also extensively studied using different assays (DPPH, ABTS, and CUPRAC). The results are presented in Table 3. DPPH and ABTS are widely used for the fast screening of the antioxidant activity of plant extracts. Meanwhile, CUPRAC, an electronic transfer-based antioxidant assessment method, stands out due to several notable advantages. These include the assay’s realistic pH close to the physiological pH, favorable redox potential, accessibility and stability of the reagents, and applicability not only to water-soluble but also to fat-soluble antioxidants. This versatility makes CUPRAC particularly suitable for analyzing various types of antioxidants present in foods, plants, or biological samples in combination with other techniques [36]. In this work, OPE exhibited relatively high antioxidant activity compared to similar literature reports. Martínez-Patiño et al. [1] reported that OPE exhibited 56.7 and 139.1 mg TE per g of extract using DPPH and ABTS assays, respectively. In the study of Goldsmith et al. [5], the antioxidant activity of OPE was 73.54 ± 2.54 mg TE per g of dried material (CUPRAC), while the corresponding value for acetone OPE (this study) was higher by approximately 29.8%. Furthermore, CUPRAC showcased statistically significant differences among the antioxidant activity of acetone and ethanolic OPE recovered under the same experimental conditions. 

The antimicrobial activity of olive extracts has been also highlighted by many studies [37,38,39]. The ability of those extracts to inhibit the growth of various bacteria has been associated with the polyphenol-rich content of olive by-products and specific phenolic compounds, such as hydroxytyrosol and oleuropein [8,40]. As shown in Table 4, the MIC and MBC values were recorded for the optimized acetone extract, hydroxytyrosol (positive control), and ethanolic OPE against Gram-negative and Gram-positive model bacteria using the microdilution method. The acetone OPE exhibited relatively lower MIC and MBC values in contrast to most literature reports. In a corresponding study by Nunes et al. [37], the effect of four extracts from solid olive waste on the growth of *E. coli* was studied. MIC concentrations of the four extracts ranged between 62.5 and 125 mg mL^−1^. In a similar study, the inhibitory effect of olive leaf extracts on the growth of *E. coli* was investigated. UAE and microwave-assisted water extraction were performed. The microwave extract showed 100% inhibition on *E. coli* growth at a concentration of 50 mg mL^−1^, while at the same concentration, the inhibition of the extract obtained from UAE was 80.9% [38]. Šimat et al. [41] evaluated the antimicrobial activity of hydroethanolic leaf extracts from six Mediterranean olive cultivars which showed MIC and MBC values of above 8 mg mL^−1^ against *E. coli*. Sánchez-Gutiérrez et al. [42] examined the antimicrobial activity of different extracts from olive leaves against a food pathogen *E. coli* strain and reported MIC values of above 30 mg mL^−1^ and MBC values of above 40 mg mL^−1^. However, Edziri et al. [43] reported that methanol extracts from four Tunisian olive cultivars exhibited significant antimicrobial activity against *E. coli* and *B. subtilis* strains. More particularly, the MIC values ranged between 32 and 128 μg mL^−1^ and the MBC values between 64 and 128 μg mL^−1^. The authors attributed the relatively low active concentrations required to provoke inhibition growth on the tested bacteria to differences in the olives’ origin and species compared to other studies. Regarding our study, acetone OPE provoked inhibition growth at lower doses compared to ethanolic OPE. This result can be directly correlated with the different phenolic compound contents of the two extracts (Table 1 and Table 2). According to the existing and previous data, the antimicrobial activity of olive by-product extracts is attributed to their phenolic profile. The proven antioxidant and antimicrobial activity of the extracts highlights their potential use in the food industry, e.g., as additives in active packaging films. More specifically, hydroxytyrosol has shown bactericidal activity against model (Table 4), food pathogen, or other bacterial strains of scientific interest. Moreover, this compound is suitable for consumption according to the EFSA’s established permitted daily dose. Taking these results into account, acetone OPE was further tested as an active ingredient of packaging films focusing on the enhancement of their biological activities.

### 2.3. Preparation and Characterization of OPE-Loaded CHT/PVA Films

**Scheme 1:**
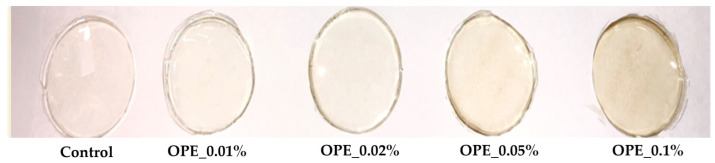
Visual appearance of CHT/PVA (control) and CHT/PVA/OPE (0.01–0.1%, *w*/*v*) films.

Recently, numerous endeavors have been made to develop bio-based packaging films with natural additives as active ingredients to tackle oxidation, degradation, and foodborne pathogens [11,16,44]. CHT and PVA are among the widely reported case studies due to their mechanical characteristics and advantages in combination or individually. Meanwhile, extracts from olive by-products have been reported to have been incorporated in CHT, PVA, or other polysaccharide films, enhancing their bioactivities [10,45,46,47]. However, the exploitation of acetone OPE as an antioxidant/antibacterial agent of CHT/PVA films has not yet been reported. In this respect, CHT/PVA (1:2, *v*/*v*) films were prepared in the absence and the presence of increasing concentrations of acetone OPE (0.01–0.1%, *w*/*v*) using the casting method. Glycerol was used as a plasticizer due to the elastic and transparent attributes that are provided to the prepared films [48]. The films appeared yellowish to brown and homogenous, depending on the concentration of OPE (Scheme 1). The main scope of this study was to investigate the biological and optical properties of CHT/PVA and CHT/PVA/OPE films, and also their basic physical characteristics.

#### 2.3.1. Determination of Physical Properties

The thickness of active packaging films is a critical parameter that affects various properties such as the physical characteristics, oxygen permeability, optical and thermal properties, and overall effectiveness in preserving food products. The medium thickness of CHT/PVA films did not differ significantly, showing that OPE loadings did not affect the overall mass (Table 5). The thickness of active coatings and edible films is typically lower than 0.3 mm [44]; thus, the CHT/PVA/OPE film values are within this range. Similar thickness values were obtained for corn starch-based films (1.5% *w*/*v* with 0.5 wt % glycerol and olive extract up to 0.2 wt %) [45]. However, da Rosa et al. [49] reported that the incorporation of olive leaf extract notably increased carrageenan film thickness. Martiny et al. [46] also observed that the thickness of carrageenan films was increased due to the addition of high olive leaf extract concentrations because of the higher mass. It is evident that the percentage of the extract loading mainly affects the thickness of the prepared films compared to the control sample (without extract).

The moisture content refers to the amount of water present in the prepared films and depends on the polymers’ composition and the drying process. For instance, Srinivasa et al. [50,51] reported that the composition of the CHT/PVA blend affected the moisture content of the films, showing that as the PVA quantity increased, so did the moisture content. As depicted in Table 5, the moisture content of CHT/PVA films was approximately 15.5 ± 0.4%, which is comparable to other research works [52]. OPE loadings did not show a significant effect on the moisture content of the prepared films at the tested concentrations. Athanasiou et al. [53] have also reported that the addition of antioxidant polymer products recovered from wine lees extract did not alter the moisture content of chitosan films. However, Annu et al. [52] reported that the moisture content of CHT/PVA films with a natural extract from *Ocimum tenuiflorum* decreased as the concentration of the extract increased (*v*/*v*). Thus, the nature of the additive (hydrophilic or hydrophobic) and the specific interactions developed with the polymer matrix seem to drive this physical attribute.

Water solubility is a key aspect considering the preparation of edible and biodegradable bio-based films. The incorporation of OPE in CHT/PVA films affected the water solubility only at the highest loading (0.1%) (*p* < 0.05) (Table 5). It can be assumed that OPE increased the hygroscopic properties of CHT/PVA films [11]. Nouri and Nafchi [54] also showed that betel leaf extract enhanced the water solubility of starch films, possibly due to the increased content of hydroxyl groups. Moreover, the initial high water solubility of CHT/PVA films (29.2 ± 0.3%) can be ascribed to the hydrophilic nature of both polymers and the addition of glycerol, as reported before [10,13]. In general, high solubility is also an indicator of biodegradability which could be an advantage for food packaging applications [55]. On the other hand, the shelf-life of packaging materials depends on their water resistance. The water solubility values of CHT/PVA/OPE films are lower compared to previous works, which can be ascribed to the preparation process [52,55].

#### 2.3.2. Optical Properties

**Figure 4:**
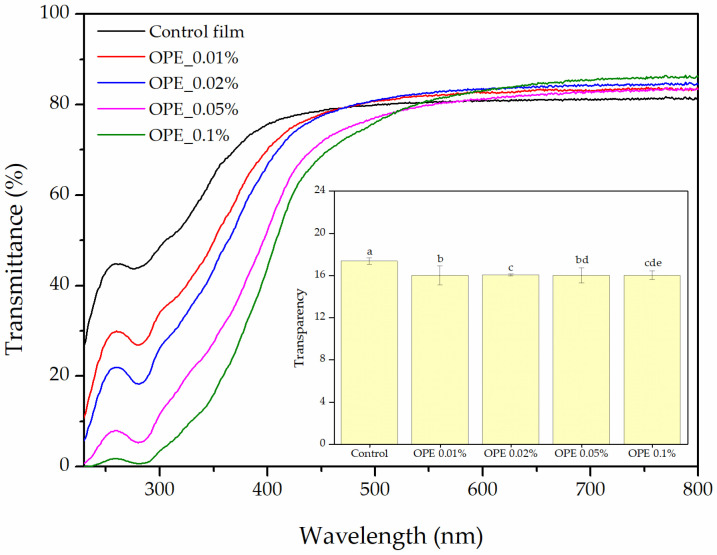
UV-Vis light transmittance of CHT/PVA and CHT/PVA/OPE films (insert graph depicts transparency measurements at 600 nm). Different lowercase letters indicate differences in significance.

Light transmittance is strongly influenced by the composition of the films, e.g., polymer material, composition of blending and incorporation of additives. Generally, extracts block UV light because of the absorption of UV radiation (the existence of hydroxyl groups of phenolic compounds). This attribute is crucial for food applications and the preservation of light-sensitive compounds, e.g., in fruits, vegetables, and meat, from oxidation and discoloration [56]. The optical properties of CHT/PVA and CHT/PVA/OPE films were determined by monitoring the transmittance in the range 220–800 nm and the transparency at 600 nm. CHT/PVA/OPE samples exhibited lower transmittance in the UV light than the control sample. The effect was dose-dependent. More specifically, the highest OPE loading (0.1%) presented the lowest transmittance in the range 220–400 nm. As shown in Figure 4, UV-C light and UV-B light were fully blocked, exhibiting approximately 0% light transmittance. Li et al. [57] observed a similar pattern using different concentrations of tannic acid in sodium alginate films. These findings were also in correlation with Kanatt et al. who reported that the incorporation of aqueous mint extract/pomegranate peel extract improved the light barrier properties of CHT/PVA films [16]. Therefore, CHT/PVA/OPE films seem to be useful UV-blocking materials that can extend the shelf-life of foods. Meanwhile, the transparency was not significantly affected. The values of the CHT/PVA/OPE films slightly decreased compared to the CHT/PVA film. As reported by Annu et al. [52], the transparency values comply with those of commercial synthetic films such as polyethylene (86.9%) and polyester (83.5%).

#### 2.3.3. Antioxidant and Antimicrobial Activities of Films

**Figure 5:**
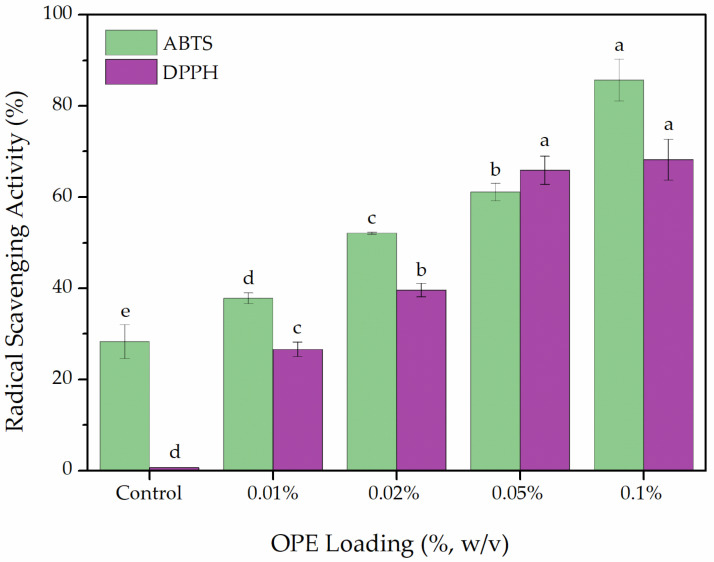
Antioxidant activity of CHT/PVA and CHT/PVA/OPE films. Different lowercase letters indicate differences in significance.

The biological activities of CHT/PVA and CHT/PVA/OPE films were also evaluated. The bio-functionalities of polymeric films with extracts from by-products are of the utmost importance. The sector of biodegradable films is ever increasing, with a compound annual growth rate of >24% (GlobalData) [58]. Different extracts have been utilized as natural preservatives of films. Nowadays, this strategy is well known and can substitute the direct incorporation of preservatives in packaged foods. Antioxidant compounds that are present in plant-based extracts tackle the oxidation process prolonging the shelf-life of lipids and fats [59]. In this respect, the antioxidant activity of CHT/PVA and CHT/PVA/OPE films was explored using DPPH and ABTS scavenging assays. As expected, CHT/PVA films did not exhibit considerable radical scavenging capacity (Figure 5); however, the incorporation of OPE resulted in significantly increased antioxidant activity (*p* < 0.05), which was dose-dependent. At the highest OPE concentration (0.1%), the DPPH^•^ and ABTS^•+^ scavenging activities of the films reached 68.2 and 85.7%, respectively. The antioxidant activity was proportional to the concentration added. Da Rosa et al. reported the same trend. As the concentration of olive leaf (50–200 mg) increased, so did the antioxidant activity of carrageenan films [49]. Moreover, the scavenging activities of CHT/PVA films loaded with a natural extract of *Ocimum tenuiflorum* were around 41.1 ± 1.17% at the highest concentration [52]. Seaweed extracts have also increased the antioxidant activity of edible films [60]. Wu et al. [61] reported that tea polyphenols, mainly flavonoids, enhanced the antioxidant activity of pomelo peel flours. Phenolic compounds scavenge free radicals due to their hydrogen-donating ability. In our study, the antioxidant activity of the enriched films can be ascribed to the main phenolic compounds of OPE, i.e., hydroxytyrosol, tyrosol, catechin, luteolin, etc. (Table 1). 

Enriched packaging films with plant-based extracts can also inhibit the growth of bacteria. The spoilage and deterioration of food products seem to be prevented by phytochemical compounds present in extracts. For instance, hydroxytyrosol or enriched extracts have shown strong antibacterial activity, as already shown in Table 3 and previous studies [8]. CHT/PVA and CHT/PVA/OPE were tested for their antimicrobial activity against two potentially pathogenic bacteria. Table 6 displays the antibacterial activity of the films evaluated as viable cell survival after 24 h. It is worth noting that *E. coli* growth was inhibited using either CHT/PVA or CHT/PVA/OPE films without differences between the enriched samples. The inherent strong antibacterial activity of CHT against *E. coli* is well known and is ascribed to its positively charged amine groups [11]. However, regarding *B. subtilis*, CHT/PVA/OPE showed dose-dependent antibacterial activity, which was also significantly increased compared to the control film (*p* < 0.05). Ultee et al. [62] have correlated the active hydroxyl groups of phenolic compounds with the ability of natural extracts to delay bacteria growth. A dose-dependent pattern against pathogenic bacteria was also observed by Musella et al., who prepared chitosan films enriched with olive leaf extract [10]. Other studies that have focused on the preservation of meat quality from bacteria contamination have utilized olive leaf extract-enriched chitosan films and aqueous mint extract/pomegranate extract-enriched CHT/PVA films [12,16]. The former showed potent antibacterial activity against Gram-negative and Gram-positive bacteria with significant susceptibility to *E. coli* strains, while the latter presented notable inhibition on the growth of Gram-positive pathogenic bacteria. To summarize, extracts from agro-industrial by-products preserve their antioxidant and antimicrobial activities as packaging film additives. Therefore, we can assume that extract-enriched films can substitute commercial additives to prevent post-packaging contamination. 

## 3. Materials and Methods

### 3.1. Materials

Olive pomace was kindly supplied by STYMON Natural Products IKE located in Patra, Greece. Folin–Ciocâlteu reagent, 2,2′-azino-bis (3-ethylbenzothiazoline-6-sulphonic acid) diammonium salt (ABTS), 2,2-diphenyl-1-picrylhydrazyl (DPPH), chitosan (from crab shells, 85% deacetylated), poly(vinyl alcohol) (average MW 13,000–23,000, 98% hydrolyzed), ammonium acetate, copper chloride dihydrate, neocuproine, p-hydroxybenzoic acid, ferulic acid, caffeic acid, syringic acid, p-coumaric acid, catechol, catechin, and acetic acid (99.8%) were purchased from Sigma- Aldrich (St. Louis, MO, USA). Potassium persulfate was purchased from Merck KGaA (Darmstadt, Germany). Potassium acetate and aluminum chloride hexahydrate were purchased from Fluka BioChemika (Buchs, Switzerland). Hydroxytyrosol, tyrosol, oleuropein, vanillyl alcohol, homovanillyl alcohol, luteolin, apigenin, and quercetin were purchased from Carbosynth (Compton, Berkshire, UK). Gallic acid hydrate and 6-hydroxy-2,5,7,8-tetramethylchroman-2-carboxylic acid (Trolox) were obtained from Tokyo Chemical Industry Co., Ltd. (Tokyo, Japan). Ethanol (99.8%) and sodium carbonate were purchased from Honeywell Specialty Chemicals (Seelze, Germany). Acetonitrile, acetone, ethyl acetate, water, methanol (≥98%, HPLC grade), and glycerol (0.5% max water content) were purchased from Fisher Scientific Corporation (Loughborough, UK). Double distilled water was used to prepare buffers and solutions.

*E. coli* strain BL21(DE3) and *B. subtilis* were taken from cultural collections of the Department of Biological Applications and Technologies and the Department of Chemistry (Ioannina, Greece). The strains were recovered from cryo-preservation and grown on Luria Bertani (LB) medium, Lennox formulation (NEOGEN Co. 620 Lesher Place, Lansing, MI 48912 USA) at 37 ± 1 °C under shaking at 160 rpm (MRC Laboratory Shaker Incubator). Agar plates were prepared with LB agar (NEOGEN Co. 620 Lesher Place, Lansing, MI 48912 USA) according to the manufacturer’s guidelines.

### 3.2. Methods

#### 3.2.1. Ultrasound-Assisted Extraction of Polyphenols from OP

The UAE of polyphenols from OP was conducted on an ultrasound water bath operating in continuous mode (120 W, 37 kHz) for 30 min (Elmasonic Easy 20H, Elma Schmidbauer GmbH, Singen, Germany). One gram of milled OP was immersed in 20 mL of solvent. The following experimental conditions were evaluated: solvent (water, ethyl acetate, ethanol, ethanol–water mixtures, acetone and acetone–water mixtures), organic solvent-to-water ratio (0:100–100:0) and solvent-to-solid ratio (5:1–40:1). The extracts were filtered through Whatman filter paper, and the supernatants were collected and centrifuged at 9000 rpm for 10 min at 7 °C (Heraeus™ Megafuge™ 8R, Thermo Fisher Scientific, Waltham, MA, USA). The organic solvent was evaporated at 35 °C using a rotary evaporator (R-114RE B-480, Buchi, Flawil, Switzerland) and water was eliminated using a lyophilizer (FDL-10N-50-TD, MRC, Tel Aviv, Israel). The obtained solid extracts (olive pomace extracts, OPEs) were stored at −20 °C for further experiments. Further optimization of the UAE process of polyphenols from OP was carried out using the ATPIO XO-SM50 ultrasound and microwave collaborative system (Nanjing Xianou Instruments Manufacture Co., Ltd., Nanjing, China). The acetone–water mixture at a ratio of 50:50 and solvent-to-solid ratio of 20:1 were kept constant. The system, equipped with a 6 mm probe, was operated in continuous mode (200 W, 37 kHz) under stirring. The effect of the extraction time (10–40 min) was evaluated. The next steps were similar to those described above. 

#### 3.2.2. Determination of Total Phenolic Content 

The total phenolic content (TPC) of the extracts was measured spectrophotometrically based on the Folin–Ciocalteu’s colorimetric assay, as described by Spyrou et al. [63], adjusting the reaction solution to a final volume of 200 μL. Briefly, each sample was prepared in the appropriate solvent at a final concentration of 1 mg mL^−1^. In a 96-well Elisa plate, 160 μL of double distilled water, 10 μL of the sample, and 10 μL of the Folin–Ciocalteu’s phenol reagent were mixed, and the plate was left for 3 min at room temperature. Then, 20 μL of saturated Na_2_CO_3_ solution (20%, *w/v*) was added to the samples and the plate was incubated for 1 h in the dark. The absorbance was measured at 725 nm (Multiskan SkyHigh, Thermo Fisher Scientific, Cleveland, OH, USA). The results were expressed as mg of gallic acid equivalents per g of dry OPE (mg GAE g^−1^ dry OPE) or per g of initial weight (mg GAE g^−1^ initial OP weight), using a gallic acid (μg mL^−1^) standard curve (y = 0.0408x − 0.037, R^2^ = 0.9943). All experiments were performed in triplicate.

#### 3.2.3. Determination of Total Flavonoid Content

The total flavonoid content (TFC) was determined spectrophotometrically according to Farasat et al. [64]. Briefly, each sample was prepared in the appropriate solvent at a final concentration of 1 mg mL^−1^. In a 96-well Elisa plate, 20 µL of each extract was mixed with 20 µL of 10% aluminium chloride, 20 µL of 1 M potassium acetate, and 180 µL of double distilled water and left at room temperature for 30 min. The absorbance was recorded at 415 nm (Multiskan SkyHigh, Thermo Fisher Scientific, Cleveland, OH, USA). The results were expressed as mg of quercetin equivalents per g of dry OPE (mg QUE g^−1^ dry OPE), using a quercetin (μg mL^−1^) standard curve (y = 0.0215x + 0.089, R^2^ = 0.9756). All experiments were performed in triplicate.

#### 3.2.4. Antioxidant Activity Assays

##### DPPH Radical Scavenging Activity

The antioxidant activity of the OPE samples was evaluated based on the ability of polyphenols to donate hydrogen to the stable free radical, DPPH^•^, in methanolic solution. The method was carried out according to Spyrou et al. [63]. Briefly, extract samples (10–100 μg mL^−1^) were mixed with 0.1 mM of DPPH methanolic solution. The mixtures were shaken vigorously and incubated for 30 min in the dark. The absorbance was measured at 517 nm using a UV/Vis microplate reader (Multiskan SkyHigh, Thermo Fisher Scientific, Cleveland, OH, USA). The results were expressed as mg of trolox equivalents per g of dry OPE (mg TE g^−1^ dry OPE), using a trolox (μg mL^−1^) standard curve (y = −0.1157x, R^2^ = 0.9965). All experiments were performed in triplicate.

##### ABTS Radical Scavenging Ability

The TEAC (Trolox equivalent antioxidant activity) assay is based on the scavenging of the free radical, ABTS^•+^, in an aqueous solution [65]. Briefly, an aqueous solution containing 7 mM ABTS and 2.45 mM potassium persulfate was prepared and allowed to react at room temperature for 16 h under darkness to obtain a stable ABTS cation radical solution. Before use, the ABTS^+^ solution was diluted with water to achieve a final absorbance of 0.7 ± 0.05 in the reaction mixture. On a 96-well Elisa plate, 270 μL of diluted ABTS^+^ solution was mixed with 30 μL of OPE sample (1 mg mL^−1^) and incubated for 30 min in darkness. The decrease in absorbance at 734 nm was measured using a UV/Vis microplate reader (Multiskan SkyHigh, Thermo Fisher Scientific, Cleveland, OH, USA). The results were expressed as mg of trolox equivalents per g of dry OPE (mg TE g^−1^ dry OPE), using a trolox (μg mL^−1^) standard curve (y = −0.0953x + 0.8517, R^2^ = 0.9932). All experiments were performed in triplicate.

##### Cupric Reducing Antioxidant Capacity Assay

CUPric Reducing Antioxidant Capacity (CUPRAC) is an electron transfer-based method. The assay was adapted to a final volume of 287 μL and performed as described by Özyürek et al. [36]. Each sample was prepared in the appropriate solvent at a final concentration of 1 mg mL^−1^. The absorbance was measured at 450 nm using a UV/Vis microplate reader (Multiskan SkyHigh, Thermo Fisher Scientific, Cleveland, OH, USA). The results were expressed as mg of trolox equivalents per g of dry OPE (mg TE g^−1^ dry OPE), using a trolox (μg mL^−1^) standard curve (y = 0.0461, R^2^ = 0.9996). All experiments were performed in triplicate.

#### 3.2.5. High-Performance Liquid Chromatography Analysis

A high-performance liquid chromatography (HPLC) system (Shimadzu, Tokyo, Japan) equipped with a photodiode array detector was used for the quantification of OPE polyphenols. A Kinetex Evo C18 reversed-phase column (5 μm, 250 × 4.6 mm) protected by a pre-column, Gemini-NX C18 (4 × 3.0 mm) (Phenomenex, Torrance, CA, USA), was used for the characterization and quantification of the total phenolic compounds of the optimized OPE. A standard method was reported before by Spyrou et al. [63]. Polyphenols were identified based on the retention times and absorption profile spectra of the standard compounds. The quantification of polyphenols was based on calibration curves at their absorption maxima (λ_max_).

#### 3.2.6. Determination of Minimum Inhibitory and Minimum Bactericidal Concentration 

The minimum inhibitory concentration (MIC) of the optimized OPE was determined by the micro broth dilution method according to the Clinical and Laboratory Standards Institute (CLSI) regulation [66]. Stock solutions of OPE were prepared in LB broth to a final concentration of 40 mg mL^−1^ and serial 2-fold dilutions were made in the same medium in 96-well sterile microplates. Freshly prepared bacterial suspensions of *E. coli* and *B. subtilis* were adjusted to 0.5 McFarland standards and then diluted properly (1:150). From these suspensions, 50 μL was inoculated into each well, which also contained 50 μL of extract to give a final bacterial concentration of approximately 5 × 10^5^ colony-forming units (CFUs) mL^−1^. A sterility control well and a growth control well were also studied for each strain. The microplates were incubated at 37 °C for 24 h. Next, the MIC values were visually and spectrophotometrically (OD_600_) determined. The lowest concentration of extract displaying no visible growth was recorded as the MIC. Hydroxytyrosol and ampicillin were used as positive controls. The determination of the minimum bactericidal concentration (MBC) was recorded as the lowest extract concentration required to kill bacteria after 24 h of incubation at 37 °C [67]. Moreover, 100 μL was taken from one well above the MIC value and spread on LB agar plates. The number of colonies was counted after 24 h of incubation at 37 °C. The concentration of the sample that led to no colony formation was considered as the MBC value. All experiments were performed in triplicate. 

#### 3.2.7. Preparation of Polymeric Films

The casting method was used to prepare bioactive films according to previous work with some modifications [16]. CHT solution (1%, *w/v*) was prepared by dissolving the biopolymer in 1% (*v*/*v*) aqueous acetic acid solution at 70 °C and 140 rpm. PVA solution (5%, *w/v*) was prepared by dissolving the synthetic polymer in ddH_2_O at 70 °C and 140 rpm. Then, centrifugation at 9000 rpm and at 20 °C for 10 min was conducted to eliminate undissolved particles. Subsequently, CHT and PVA solutions were mixed at a ratio of 1:2. Glycerol (1%, *v*/*v* of the final film solution) was used as a plasticizer. OPE was employed as a bioactive component/additive at a range of concentrations (0.01, 0.02, 0.05, and 0.1%, *w/v* of the final film solution). The final mixtures were blended and degassed for 15 min at 30 °C in a sonication bath operating in continuous mode (120 W, 37 kHz) (Elmasonic Easy 20H, Elma Schmidbauer GmbH, Singen, Germany). Finally, the blended solutions were poured into Petri dishes (88 mm, inner diameter) and dried at 45 °C, O/N before peeling off. A control film without additives was also prepared. Film thickness was determined in five spots on each strip by using a digital micrometer (OEM LD001, Athens, Greece, sensitivity 0.001 mm). CHT/PVA is the abbreviation for the control film, and CHT/PVA/OPE is the abbreviation for the enriched films with different loadings.

#### 3.2.8. Characterization of CHT/PVA and CHT/PVA/OPE Films

Optical Properties

The UV–visible spectra of CHT/PVA and CHT/PVA/OPE films were obtained by a UV spectrophotometer (Agilent Cary 60, Santa Clara, CA, USA) at a wavelength range of 200–800 nm. The film samples were cut equally and placed in the spectrophotometer test cell. The air was used as a reference. The film transparency was calculated by using Equation (1) [16,57,68]. Each sample was measured in triplicate.

(1) $$Transparency=\frac{{log\;\%T}_{600}}{Y}$$

where T_600_ represents the transmittance at 600 nm and Y is the thickness (mm) of the film. 

##### Moisture Content and Water Solubility

The moisture content and the water solubility of the CHT/PVA and CHT/PVA/OPE films were investigated and estimated as reported by Athanasiou et al. [53]. 

#### 3.2.9. Antioxidant Activity of Films

The antioxidant activity of the CHT/PVA and CHT/PVA/OPE films was examined by DPPH and ABTS assays as described before with minor modifications [69]. A solution of 0.1 mM DPPH^•^ in ethanol was prepared. For this study, 10 mg of the different films (approximately 1 cm^2^) were placed in Eppendorf tubes, in which 300 μL of the DPPH^•^ solution and 700 μL of ethanol were added to reach a final volume of 1 mL. The mixtures were stirred vigorously and kept in the dark at room temperature for 30 min. The absorbance was measured spectrophotometrically at 517 nm. ABTS^•+^ solution was prepared as described in Section 3.2.4. For this study, 2 mg of the different films (approximately 0.25 cm^2^) were added to 1 mL of the ABTS^•+^ solution. The mixtures were stirred vigorously and kept in the dark at room temperature for 30 min. The absorbance was measured spectrophotometrically at 734 nm. The antioxidant activity of the CHT/PVA and CHT/PVA/OPE films was determined according to Equation (2) as follows:

(2) $$Antioxidant\;activity\;(\%)=\frac{A_{control}-A_{sample}}{A_{control}}\times 100$$

where Acontrol is the absorbance of the control(t^0^), and Asample is the absorbance of the different film samples(t_30_).

#### 3.2.10. Antibacterial Activity of Films

The antibacterial activity of the CHT/PVA and CHT/PVA/OPE films was evaluated as viable cell survival on the surface of the films as described by previous works [70,71,72]. A fresh bacterial suspension of *E. coli* and *B. subtilis* was adjusted to 0.5 McFarland standards and then diluted properly (1:10 or 1:100, depending on the bacterium). Aliquots of 10 μL of diluted bacterial inoculum containing 10^6^ CFu mL^−1^ were aseptically applied as a standing droplet on the surface of the films (0.5 × 0.5 cm) and incubated at 37 °C for 24 h. Then, the films were excessively washed with phosphate buffer saline (1:100). Subsequently, the supernatants were properly diluted (1:100 or 1:1000, depending on the strain) with phosphate buffer saline. Finally, 100 μL from these solutions was transferred to LB agar plates and spread uniformly. After 24 h of incubation at 37 °C, the number of colonies formed was counted. A positive control without a film was also studied. The viable cell number was expressed as colony-forming units per milliliter (CFUmL^−1^). More specifically, 10^1^ CFu mL^−1^ for *E. coli* and 10^4^ CFu mL^−1^ for *B. subtilis*.

#### 3.2.11. Statistical Analysis 

All analyses were carried out in triplicate and the results were recorded as mean ± standard deviation. Student’s t-test, one-way ANOVA analysis and Tukey’s multiple comparison test were carried out using IBM SPSS Statistics version 21 (SPSS Inc., Chicago, IL, USA) to compare the mean values of each treatment and to determine the statistical significance (*p* < 0.05).

## 4. Conclusions

To conclude, UAE was effectively used for the recovery of polyphenols from OP using a single-factor experiment approach. The results of studying the four independent variables showcased that the acetone–water (50%) mixture, the 1:20 solid-to-liquid ratio, and 20 min of extraction time yielded the highest TPC, TFC, hydroxytyrosol, tyrosol, luteolin, and catechin values. Acetone OPE also exhibited high antioxidant/antiradical and antimicrobial activities. Therefore, it is evident that UAE using acetone promotes the extractability of phenolic compounds of interest from OP, reaching high yields compared to similar studies. For these reasons, CHT/PVA films were prepared with different OPE loadings (%, *w/v*) as a possible application. The incorporation of OPE did not affected the physical properties of the films. The water solubility of CHT/PVA/OPE films was slightly increased at the highest OPE loading (0.1%, *w/v*), which is also an indicator of biodegradability. On the other hand, the antioxidant capacity, the UV-blocking ability, and the antibacterial activity were notably enhanced. Furthermore, it is important to note that in vitro biocompatibility studies are currently underway regarding OPE and CHT/PVA/OPE films in common cell lines to meet food safety standard requirements.

This work provides a simple and efficient strategy to acquire polyphenolic extracts rich in hydroxytyrosol from an agro-industrial by-product, which is a target antioxidant compound, with special attention given to its application as an active ingredient of packaging films. The practical applications of OPE are numerous, covering the food, nutraceutical, and cosmetic sectors; hence, extraction techniques such as UAE broaden its exploitation. Further efforts should be realized on the in vivo biocompatibility of these products and possible approaches to commercialization. 

## Figures and Tables

**Table 1:** HPLC profile of the acetone and ethanolic extracts from olive pomace.

Bioactive Compound	λ_max_ (nm)	Concentration (mg g^−1^ Dry Acetone OPE)	Concentration (mg g^−1^ Initial dw) ^α^	Concentration (mg g^−1^ Dry Ethanolic OPE)
Gallic acid	270	0.39 ± 0.03	0.1	n.d.
p-Coumaric acid	310	0.21 ± 0.12	0.05	0.18 ± 0.02
Caffeic acid	320	0.19 ± 0.04	0.05	0.33 ± 0.02
Ferulic acid	320	0.07 ± 0.01	0.02	n.d.
Vanillic acid	260	1.13 ± 0.07	0.29	1.19 ± 0.02
Hydroxytyrosol	280	48.47 ± 0.13	12.46	43.02 ± 1.6
Homovanillyl alcohol	280	0.19 ± 0.10	0.05	n.d.
Tyrosol	280	6.82 ± 0.07	1.75	5.73 ± 0.55
Vanillyl alcohol	280	1.29 ± 0.11	0.33	0.89 ± 0.69
Luteolin	346	2.70 ± 0.18	0.69	2.35 ± 0.08
Catechin	280	3.93 ± 0.92	1.01	3.48 ± 2.18
Apigenin	336	0.97 ± 0.04	0.25	0.86 ± 0.06
p-Hydroxybenzoic acid	255	0.52 ± 0.06	0.13	1.02 ± 0.3

^α^ Standard error was ≤0.001. Refers to the initial dw from the acetone extraction.

**Table 2:** Characterization of the optimized acetone and ethanolic extracts from OP. Asterisks indicate statistically significant differences between the two samples (* *p* < 0.05, ns: not significant).

OPE	TPC (mg GAE g^−1^ Dry OPE)	TPC (mg GAE g^−1^ dw)	TFC (mg QE g^−1^ Dry OPE)	TFC (mg QE g^−1^ dw)	Extraction Yield (%)
Acetone 50%	168.8 ± 5.5 ^ns^	43.5 ± 1.5 ^ns^	10.3 ± 0.4 *	2.7 ± 0.3 ^ns^	25.8 ± 0.3% *
Ethanol 50%	165.2 ± 0.8	38.1 ± 3.5	7.6 ± 0.8	1.8 ± 0.3	23.1 ± 0.5%

**Table 3:** Summary of antioxidant activity of the optimized acetone and ethanolic extracts from OP. Asterisks indicate statistically significant differences between the two samples (* *p* < 0.05, ns: not significant).

OPE	DPPH (mg TE g^−1^ Dry OPE)	ABTS (mg TE g^−1^ Dry OPE)	CUPRAC (mg TE g^−1^ Dry OPE)
Acetone 50%	413.6 ± 1.9 *	162.7 ± 3.4 ^ns^	384.9 ± 7.9 *
Ethanol 50%	406.8 ± 2.4	173.3 ± 5.2	351.9 ± 2.4

**Table 4:** MIC and MBC values of acetone OPE, ethanolic OPE, and hydroxytyrosol against Gram-positive and Gram-negative model bacteria.

Sample	*E. coli*		*B. subtilis*	
	MIC (mg mL^−1^)	ΜBC (mg mL^−1^)	MIC (mg mL^−1^)	ΜBC (mg mL^−1^)
Acetone 50%	2.5	5	10	20
Ethanol 50%	5	10	10	40
Hydroxytyrosol (positive control)	0.3	0.6	1	2

**Table 5:** Physical properties of CHT/PVA and CHT/PVA/OPE films. Different lowercase letters indicate differences in significance.

CHT/PVA Films	Moisture Content (%)	Water Solubility (%)	Thickness (mm)
Control	15.5 ± 0.4 ^a^	29.2 ± 0.3 ^b^	0.11 ± 0.01 ^a^
OPE_0.01%	16.8 ± 0.3 ^a^	28.1 ± 0.6 ^b^	0.12 ± 0.02 ^a^
OPE_0.02%	16.6 ± 0.4 ^a^	29.7± 0.4 ^b^	0.12 ± 0.01 ^a^
OPE_0.05%	16.2 ± 0.7 ^a^	29.8 ± 0.1 ^b^	0.13 ± 0.02 ^a^
OPE_0.1%	17.5 ± 0.5 ^a^	32.7 ± 0.5 ^a^	0.12 ± 0.01 ^a^

**Table 6:** Antibacterial activity of CHT/PVA and CHT/PVA/OPE films. Asterisks indicate statistically significant differences between the two samples (** *p* < 0.01, *** *p* < 0.001).

Sample	*E. coli*	*B. subtilis*
	Cell Viability	
	(10^1^ CFu mL^−1^)	(10^4^ CFumL^−1^)
Control	6 ± 1	225 ± 7
OPE_0.01%	n.d. ***	168 ± 15 **
OPE_0.02%	n.d. ***	76 ± 3 ***
OPE_0.05%	n.d. ***	42 ± 4 ***
OPE_0.1%	n.d. ***	42 ± 1 ***

n.d.: not detected.

## Data Availability

Data contained within the article.
